# Use of indocyanine green for detecting sentinel lymph nodes in breast cancer: letter to the editor

**DOI:** 10.1186/s12957-017-1138-z

**Published:** 2017-03-27

**Authors:** Samir Hidar

**Affiliations:** F.Hached University Teaching Hospital, Sousse, Tunisia

**Keywords:** Breast cancer, Sentinel node, Indocyanine green

## Abstract

In a previous issue of the journal, Oldřich Coufal and Vuk Fait reported a pilot study that specifically addressed the use of indocyanine green for detecting sentinel lymph nodes in breast cancer within a European population. They concluded that fluorescence method cannot currently be considered a method fully comparable with using radioisotopes in this setting. We consider that the absence of a learning curve, the low mean of retrieved sentinel nodes, and the possibility that migration of indocyanine green occurred after the initial biopsy limit the strength of their conclusion.

Dear Sir,

We read with great interest the paper published in the journal by Oldřich Coufal and Vuk Fait entitled “Use of indocyanine green and the HyperEye system for detecting sentinel lymph nodes in breast cancer within a population of European patients: a pilot study”. The authors specifically report the results of indocyanine green (ICG) for detecting sentinel lymph nodes (SLNs) in a European population with breast cancer. The authors are to be congratulated for this work.

In the view of their results, Coufal and Fait concluded that fluorescence method using ICG cannot currently be considered a method fully comparable with using radioisotopes (RI) in European patients. Also, their study is well conducted and we have some remarks about this conclusion.

Coufal and Fait have well-recognized expertise in surgical oncology. Despite the shorter learning curve than other methods, detecting SLN using fluorescence imaging with ICG—as all other techniques in SLN—has its own learning curve independent of surgical expertise. It is not clearly mentioned if the authors performed other ICG procedures before starting the study or if they just received technical assistance from a HyerEye technician. It is difficult to compare the results of a newly implanted technique with those of a technique performed for many years.

The mean number of retrieved sentinel nodes using ICG in the abovementioned study is 1.3 per patient. This value is lower than the published data using NIR in which the mean number of nodes is generally closer to an interval between 2 and 4 per patient [1–3] even in a European population [4–6]. It is well known that a lower number of retrieved nodes is responsible of a higher false negative rates. Very interestingly, the authors demonstrated that all sentinel nodes extirpated by the sole gamma probe demonstrated ex vivo fluorescence. They should be congratulated for doing this; they concluded that the problem is that tissue permeability to fluorescence is the limitative factor and not the insufficient transport of ICG. Another explanation of the absence of fluorescence of sentinel nodes is that ICG passage occurred after the retrieval of SLN using NIR probe. It would be therefore not a question of permeability but of time interval between ICG administration and SLN biopsy.

The main merit of the study by Coufal and Fait is that it raises an important issue that can emerge when applying new techniques in a different setting of populations with different characteristics and particularly BMI in this situation. However, in our opinion its statistical power (two positive SLN/ten patients; one false negative) makes it unable to confirm or not to confirm results from previous studies.

## Abbreviations

ICG: Indocyanine green
NIR: Near infrared (fluorescence)
SLN: Sentinel lymph node
